# The Potential of Xylooligosaccharides as Prebiotics and Their Sustainable Production from Agro-Industrial by-Products

**DOI:** 10.3390/foods12142681

**Published:** 2023-07-12

**Authors:** Kim Kley Valladares-Diestra, Luciana Porto de Souza Vandenberghe, Sabrina Vieira, Luis Daniel Goyzueta-Mamani, Patricia Beatriz Gruening de Mattos, Maria Clara Manzoki, Vanete Thomaz Soccol, Carlos Ricardo Soccol

**Affiliations:** 1 Department of Bioprocess Engineering and Biotechnology, Centro Politécnico, Federal University of Paraná, Curitiba 81531-980, Paraná, Brazillvandenberghe@ufpr.br (L.P.d.S.V.);; 2 Vicerrectorado de Investigación, Universidad Católica de Santa María, Urb. San José s/n—Umacollo, Arequipa 04000, Peru

**Keywords:** xylooligosaccharides, prebiotics, lignocellulosic biomass, agro-industrial by-products

## Abstract

In recent years, concerns about a good-quality diet have increased. Food supplements such as prebiotics have great nutritional and health benefits. Within the diverse range of prebiotics, xylooligosaccharides (XOs) show high potential, presenting exceptional properties for the prevention of systemic disorders. XOs can be found in different natural sources; however, their production is limited. Lignocellulosic biomasses present a high potential as a source of raw material for the production of XOs, making the agro-industrial by-products the perfect candidates for production on an industrial scale. However, these biomasses require the application of physicochemical pretreatments to obtain XOs. Different pretreatment methodologies are discussed in terms of increasing the production of XOs and limiting the coproduction of toxic compounds. The advance in new technologies for XOs production could decrease their real cost (USD 25–50/kg) on an industrial scale and would increase the volume of market transactions in the prebiotic sector (USD 4.5 billion). In this sense, new patents and innovations are being strategically developed to expand the use of XOs as daily prebiotics.

## 1. Introduction

In recent years, the food industry has developed different types of new food products in order to obtain functional foods with greater benefits for health and nutrition. In this sense, the functional food market is proliferating to increase the value of ingredients added to food, particularly food supplements that prevent the occurrence of serious diseases that can cause public health problems [1]. These new products have been widely accepted by consumers, who demand products with lower fat, salt, and sugar content, supplemented with functional food ingredients (prebiotics and probiotics), which help in health care.

Prebiotics are widely used as nondigestible food supplements mainly due to their different health benefits for various systemic disorders such as gastrointestinal, cardiovascular, neurological, inflammatory, oncological, and endocrine systems [2]. There are different types of prebiotics, which are mostly indigestible fibers, among which low-molecular-weight oligosaccharides stand out.

Xylooligosaccharides (XOs) are nondigestible oligosaccharides composed mainly of xylose units with high prebiotic potential. Due to their physicochemical characteristics, XOs are highly resistant to gastrointestinal enzymes and gastric acids, which allows them to pass through the upper gastrointestinal tract without being digested, until they reach the lower intestine and are metabolized by probiotic bacteria, mainly from the *Bifidobacterium* and *Lactobacillus* genera. An XOs-supplemented diet promotes host health in multifaceted ways due to XOs’ immunomodulatory, anticancer, antimicrobial, growth-regulating, antioxidant, and other bioactive properties [2,3]. That is why XOs present a high demand in the food market. However, the natural extraction source of these prebiotics leads to low concentrations of them, which limits their industrial use [4]. For this reason, new sources for the extraction and production of XOs have been developed and studied, focusing on the high potential of lignocellulosic biomass as a new source of raw material. 

Lignocellulosic biomass is composed of carbohydrates and lignin polymers, where hemicellulose represents the second most abundant polysaccharide and is mainly composed of xylan [5]. In this way, lignocellulosic biomass is emerging as a high potential source for the production of XOs. Within the vast diversity of lignocellulosic biomass, agro-industrial by-products are of strong interest to the industry, due to their high production volume, worldwide distribution, and low market price. However, due to its recalcitrant nature, it is necessary to expand physicochemical pretreatments [6]. Different types of pretreatments have been used in order to obtain greater efficiency in the production of XOs from agro-industrial by-products. Traditional pretreatment methods using acid, alkaline, and high temperatures allow the release of XOs with different polymerization degrees and toxic chemical derivatives [7]. New pretreatment methods using recyclable and/or biodegradable catalysts, which allow high production efficiency without generating toxic compounds, are being developed by different study groups.

In this review, the high potential of XOs as a food supplement is shown, describing their different health benefits. Alternative sources of extraction and production of XOs are discussed, highlighting the use of agro-industrial by-products due to their high hemicellulose content, their renewable capacity, and their low price, which would enable a higher competitiveness of XOs in the market. The different pretreatment methods applied in the production of XOs and the demand of these prebiotics in the global market scenario are also shown. Finally, a discussion about the different patents developed for the production and commercialization of XOs is presented, including how innovation is facing the new challenges in technological development to consolidate XOs as a food supplement for the daily diet.

## 2. The Importance of Xylooligosaccharides as Prebiotics

### 2.1. Prebiotics

The International Scientific Association for Probiotics and Prebiotics (ISAPP) defines prebiotics as “a substrate that is selectively utilized by host microorganisms conferring a health benefit”. This definition is applicable to both humans and animals [8]. In this sense, prebiotics are indigestible food supplements that play an important role in stimulating the growth of beneficial bacteria for health. These beneficial bacteria, also known as probiotics, are part of the intestinal microbiota, with the genera *Lactobacillus* (some former *Lactobacillus* probiotics are now classified in genera *Ligilactobacillus*, *Lactiplantibacillus*, and *Limosilactobacillus*) and *Bifidobacterium* being the main beneficial probiotics [9,10,11].

In addition to increasing the levels of healthy microbiota in the gut, a diet supplemented with prebiotics increases the metabolism of probiotic bacteria, producing beneficial chemical compounds such as propionate, primary butyrate, and secondary butyrate. On the other hand, pathogenic bacteria of the *Enterobacteriaceae* family (enteric bacteria) and of the *Clostridium* genus do not have the capacity to metabolize prebiotics, avoiding their proliferation and indirectly stimulating the colonization of the intestinal epithelium by probiotics [1]. 

Nondigestible oligosaccharides are low-molecular-weight prebiotics derived from carbohydrates like polysaccharides found in plants. These prebiotics have high potential due to their great abundance and are classified according to their chemical nature. Thus, there are a wide variety of prebiotics defined by the most abundant type of monosaccharide in the main chain, such as fructooligosaccharides, galactooligosaccharides, maltooligosaccharides, isomaltooligosaccharides, and XOs [1,12,13].

### 2.2. Xylooligosaccharides as Prebiotics

XOs are nondigestible oligosaccharides that present in their main chain xylose monosaccharides of 2–10 units in size linked by β-1,4-xylosidic bonds. This type of link allows XOs to resist the attack of gastric enzymes, so they pass through the upper gastrointestinal tract without being digested, until they reach the lower intestine and are metabolized by the probiotic microbiota. The prebiotic effectiveness of XOs depends on the polymerization degree of its main chain. Smaller chains with a polymerization degree of 2–4 are particularly effective in promoting the growth of specific probiotics, especially those belonging to the *Bifidobacterium* genus. Additionally, XOs exhibit improved prebiotic properties when there is a limited presence or an absence of monosaccharides, such as glucose and xylose [5].

A characteristic of XOs is their high stability at acidic pH, with ranges of 2.5–8, which gives them the capacity to resist gastric juices. They present a high stability at temperatures above 100 °C. On the other hand, XOs have organoleptic properties of a moderate sweetness level and do not have an unpleasant taste, which allows their supplementation in any type of food, and the xylobiose has 30% of the sweetness of sucrose [14].

Arabinoxylans (AX) are xylans with arabinose substitution at the C-2 and/or C-3 positions of the xylan backbone, and are most present in cereals like wheat, barley, maize, and rice [15]. Hydrolysis of AX leads to a mixture of unsubstituted and arabinose-substituted XOs, the so-called arabinoxylan-oligosaccharides (AXOs) [16]. Each species of probiotic has specific preferences between XOs and AXOs assimilation. When in the colon, AXO and XO fragments are further degraded to xylose and arabinose by extracellular and/or intracellular arabinofuranosidases and xylosidases produced by specialized bacteria, including *Bifidobacterium* species [16]. AXO, with its arabinose side chains, has been shown to selectively stimulate the growth of bifidobacteria, whereas the effects of xylan-oligosaccharides may vary depending on their specific composition.

For example, in the human microbiota, 12 different phyla are predominant, among them Proteobacteria, Firmicutes, Actinobacteria, and Bacteroidetes [17,18]. The most well-known probiotic strains, such as Bifidobacterium (Actinobacteria) and Lactobacillus (Firmicutes), have distinct systems for utilizing (A)XOs. Cross-feeding plays a crucial role in the breakdown of intricate substrates in the gut. Strains that generate arabinofuranosidases can utilize the arabinose attachments found in AXOs. In contrast, other strains can consume the unsubstituted XOs produced. Bifidobacterium adolescentis, for example, can consume both AXOs and undecorated XOs, while Lactobacillus brevis utilizes only XOs [18]. Similarly, Weissella confusa (Firmicutes), a potential probiotic, has been found to use XOs but not AXOs [16,18].

XOs prebiotics can be consumed in many forms in food and feed (Table 1). For animals, it is normally incorporated at a given percentage in the daily diet. In the case of human consumption, it can be ingested directly as a pure supplement, as in the case of capsule intake [19], or it can be incorporated in beverages (like juices), biscuits, and breakfast cereals, among other things [20,21,22,23]. The addition of XOs in those products has great acceptance according to the literature since they contribute to sensorial properties by increasing sweetness and taste intensity. Further, the heat stability of XOs makes them suitable for baked products [24].

### 2.3. Beneficial Properties of Xylooligosaccharides

**Figure 1:**
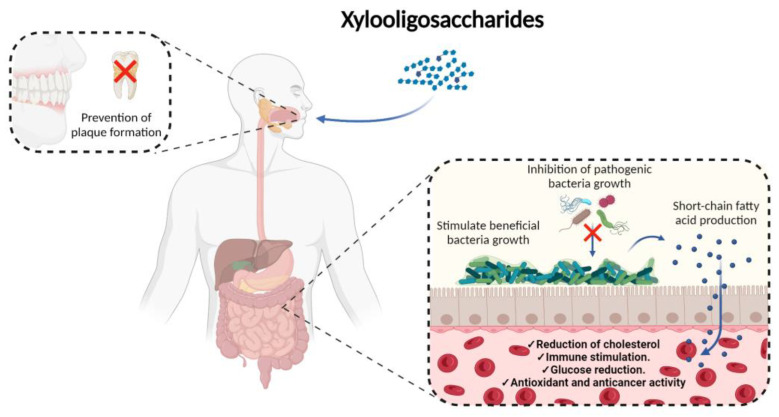
Xylooligosaccharides beneficial characteristics as prebiotic in humans.

In general, XOs increase digestion and absorption of nutrients, but also present health benefits by preventing the growth of pathogenic bacteria. The inhibition of pathogenic bacteria due to the action of XOs is mediated by two mechanisms: (i) increasing the proliferation and colonization of probiotic bacteria in the intestinal epithelium, and (ii) decreasing the pH by inducing the production of organic acids, such as lactic acid and acetic acid. Thanks to these mechanisms, XOs indirectly prevent gastrointestinal infections, maintaining fecal water levels and preventing diarrhea. In addition to suppressing the activity of enteric bacteria, they decrease the production of toxic compounds such as amines [14]. Other favorable effects of XOs are to improve the proliferation of cecal epithelial cells and prevent the formation of dental plaque (Figure 1) [30,31].

On the other hand, XOs present specific indirect actions within the intestinal tract. These prebiotics are used and metabolized by probiotic bacteria, generating an increase in the production levels of small-chain fatty acids (SCFAs), with butyrate and propionate SCFAs being the most produced. High levels of SCFAs maintain the integrity of the gastrointestinal barrier by regulating cecal cell proliferation and apoptosis, and by encouraging goblet cell differentiation via Notch and Wnt/β-catenin signaling pathways [31]. It has also been reported that dietary supplementation with XOs can increase the expression of molecular chaperones and improve the ubiquitination of proteases, which would also demonstrate that XOs have a healthy protective activity in the mammalian intestine. Finally, the SCFAs induced by the metabolization of XOs induce the activation of expression genes and the synthesis of proteins in cecal cells, and are able to suppress the expression of proinflammatory cytokines through activating host GPR109a or inhibiting histone deacetylases [31,32].

These characteristics give XOs greater potential compared to other established prebiotics such as fructooligosaccharides, galactooligosaccharides, and inulin [9,33], hence making them interesting supplementary food ingredients. Further, probiotics of the *Bifidobacterium* genus present a greater predisposition for the consumption of pentoses compared to prebiotics composed of hexoses (fructooligosaccharides and galactooligosaccharides). In addition, it has been reported that due to the high induction of responses beneficial to health, smaller daily doses of XOs are necessary in food supplementation compared to other prebiotics, with XO dietary supplementation requiring only 1.4–2.8 g per day [34,35]. This makes XOs more economically competitive than other prebiotics, which has high potential for consumer preference as a healthy food product. 

However, the natural sources for obtaining XOs such as vegetables, fruits, honey, milk, and bamboo shoots are scarce and limited [4]. In this sense, industrial-scale production seeks new sources of cheap and renewable raw materials to deal with the high demand for existing prebiotics. In recent years, lignocellulose biomasses have emerged as a source of raw material in the production of high-value-added biomolecules, including XOs prebiotics [36]. The great distribution worldwide, its great abundance, and its renewable nature make lignocellulosic biomass a natural source that is easily accessible and cheap compared to other raw materials. Within the huge set of lignocellulosic biomasses, agro-industrial residues or by-products obtain greater interest due to their high production volumes [5].

## 3. Agro-Industrial by-Products as Substrates for Xylooligosaccharides Production

### 3.1. Agro-Industrial by-Products Employed for Xylooligosaccharides Production

The cost of the production of XOs is a limiting factor, so their obtention from agro-industrial waste, including many grain by-products, has been considered a potential cost-reduction strategy as this is a low-cost, renewable, and abundant raw material [35]. Xylan, the second most abundant biopolymer in nature, constitutes most of the hemicellulose that can be degraded into XOs, using combinations of pretreatments and enzymatic hydrolysis [37]. Despite the benefits of using agro-industrial residues, their varying composition is a challenge, because depending on the chemical composition, the residues may be more or less suitable for XOs production. Residues with higher amounts of xylan and low amounts of lignin are better options. Corn cob, wheat straw, rice straw, corn stover, switchgrass, and sugarcane bagasse are some of the relevant residues for XOs production, due to their high levels of hemicellulose [35,38]. The high xylan content of corn cob is considered attractive for XOs production [39]. Xylans from agro-industrial residues are classified depending on the type of side groups and degree of substitutions. For example, arabinoxylans are generally present in sugarcane and cereals, such as wheat, rye, barley, oats, rice, corn, and sorghum, and in grasses there is a higher presence of L-arabinose [40].

### 3.2. Lignocellulosic Biomass Pretreatment

Agro-industrial by-products are mostly composed of cellulose, hemicellulose, and lignin structures. Cellulose and hemicellulose can be enzymatically hydrolyzed into glucose and xylose; however, cellulose is strongly associated with hemicelluloses and lignin, preventing the access of hydrolytic agents, and its crystalline structure is also an extra obstacle to hydrolysis [41]. To increase the efficiency of the use of lignocellulosic residues, it is necessary to perform a step before the enzymatic hydrolysis, called pretreatment, to extract xylan from the plant cell wall. Several pretreatment techniques have been reported in the literature, such as steam explosion, solvent extraction, and thermal pretreatment using acids or bases, organosolv, deep eutectic solvents, and hydrothermal pretreatment [6,42,43]. During pretreatment, hemicellulose can be depolymerized to produce various xylosugars, among them short-chain polysaccharides with different degrees of polymerization, which are considered attractive because of their potential use to promote the growth of intestinal bacteria and improve immunity [41]. Alkaline pretreatment has the disadvantage of partially degrading carbohydrates and causing equipment corrosion and environmental contamination. Acid pretreatment generates undesirable sugar monomers and many toxic by-products, such as furfural, hydroxymethylfurfural, and formic acid, and excessive degradation of xylan to xylose results in lower purity of the XOs. Hydrolysis with acetic acid has been used to prepare XOs because of its fast reaction and high yield [44]. 

Lignin and polysaccharides, such as hemicellulose and cellulose, are primarily bound together by noncovalent bonds, specifically hydrogen bonds and van der Waals forces. These bonds play a significant role in the structural integrity of plant cell walls, where lignin acts as a matrix that binds and strengthens the polysaccharide components. While noncovalent interactions are predominant, there can also be some covalent linkages between lignin and polysaccharides, such as ester bonds, which are susceptible to hydrolysis. Alkaline extraction is performed using salts at high temperatures, which leads to deacetylation of the compounds. Xylan, a type of hemicellulose, naturally presents O-acetyl groups at the hydroxyl ends of its structure, improving its solubility in water. However, during the alkaline extraction process, the acetyl groups are removed. On the other hand, extraction with water can isolate water-soluble hemicelluloses, which have high molar mass and less substitution of arabinose. In addition, this form of extraction helps in the preservation of the hemicellulose structure [1].

Acid or alkaline pretreatments can cause environmental pollution because reagents need to be discarded after the process. Alternatively, hot water pretreatment has been used with the advantage of not requiring reagents. It is based on the autoionization of water at high temperatures, producing protons that break the glycosidic bonds and generate organic acids, which can act as catalysts for the degradation of the hemicellulose and lignin fractions of the biomass [45]. Pretreatment using imidazolium brings positive results in biomass delignification, allowing the high recovery of cellulose-rich and hemicellulose-rich fractions separately. Imidazole has the advantage of low vapor pressure, high boiling point, and low toxicity, and it is recyclable and reusable [46].

Ionic liquids are solvents that are integrally composed of ions, which present organic properties to reduce the intractability of the lignin/carbohydrate complex, decreasing the recalcitrance of lignocellulosic biomass. On the other hand, deep eutectic solvents have great potential as a method to be applied in the extraction of lignocellulosic fractions, such as hemicellulose. Deep eutectic solvents generally have a component that acts as a hydrogen-bond acceptor and another component that acts as a hydrogen-bond donor, and are renewable, biodegradable, and profitable [47]. Ionic liquids and deep eutectic solvents are included within green solvents [48]. Wang et al. (2017) [49] evaluated imidazole-based ionic liquid with an anionic component (HSO_4_). It generated greater hydrolysis of xylan in the pretreatment of xylan-rich hardwood, hydrolyzing more than half of the xylan in the products as xylose or XOs. Morais et al. (2018) [50] used deep eutectic solvents (choline chloride and urea) to extract xylan from hardwood, demonstrating the successful extraction of xylan from *Eucalyptus globulus* wood.

Comparative to conventional approaches, microwave pretreatment techniques are a promising approach to increase the efficiency and speed of XOs production, providing a faster reaction rate and lower energy consumption [51]. Recently, the use of microwaves has been considered a promising heating method for the selective and controlled hydrothermal depolymerization of biomass. The energy from microwaves is absorbed by the water present in the reaction medium, directly generating heat in the substrate. This results in more efficient and faster heating compared to conventional methods. The application of microwaves during the enzymatic hydrolysis of xylan can increase the reaction rate, accelerating the breakdown of glycosidic bonds through rapid and selective heating [52]. Controlled microwave application can lead to more precise and specific enzymatic hydrolysis, preventing excessive degradation of XOs into monosaccharides. Microwave treatment causes fragmentation and swelling, leading to the degradation of lignin and hemicellulose, thereby improving pentose yield. Results have shown that microwave pretreatment is a promising method for lignocellulosic degradation of rice husks [53]. Microwave-assisted reactions significantly decrease xylose content in hydrolysates and greatly increase the yield of XOs compared to conventional heating methods [54]. Microwave irradiation treatment involves exposing lignocellulosic biomass to electromagnetic waves with wavelengths between 1 mm and 1 m and frequencies ranging from 300 to 300,000 MHz [42]. The principle of microwave heating involves rapid rotation of polar molecules, resulting in faster and more intense activation of polar species. Therefore, the use of microwave-assisted heating for reactions under hydrothermal conditions is considered a highly favorable strategy to obtain selective, nontoxic, and high-purity production of XOs from lignocellulosic biomass [55].

Microwave-assisted pretreatment generally avoids the use of solvents, auxiliary chemicals, or separating agents. Additionally, the lower chance of waste generation after the pretreatment process is recognized as an advantage of microwave-based approaches. Compared to other conventional heating approaches, microwaves reduce the process time by tenfold, resulting in lower energy consumption [56]. In an investigation of microwave-assisted acid hydrolysis of sugarcane bagasse for XOs production, response surface analysis indicated that microwave-assisted acid hydrolysis using H_2_SO_4_ at a concentration of 0.24 M for 31 min resulted in a maximum XOs yield of 290.2 mg/g. It was observed that the xylose yield increased with increasing acid concentration and residence time, and no degraded sugar products were formed during the process [57]. In a study on microwave-assisted hydrothermal depolymerization of beechwood hemicellulose aimed at producing high-purity XOs, a good balance was achieved between the net yield (81%) and the purity of XOs (96% by carbon mass) [55]. Regarding the impact of microwave-assisted deep eutectic solvent pretreatment on the production of XOs from wheat straw, the results demonstrated that this pretreatment is an efficient, fast, and promising technique for xylan extraction. However, further research is needed to develop economically viable methods for the separation and purification of the XOs extracted from the mixture [58].

In Table 2, examples of XOs produced from agro-industrial residues using different pretreatment methods are shown.

### 3.3. Enzymatic Hydrolysis for Xylooligosaccharides Production

Enzymatic methods are commonly utilized to obtain XOs, especially due to their high yields and specificity in generating products. The advantages also include the use of milder operating conditions, resulting in less generation of polluting waste. The enzymatic method can be used either with enzymes in biological treatments or applied after physicochemical pretreatment to increase XOs production from lignocellulosic biomass (Table 3) [72,73,74,75].

The group of enzymes involved in this method is called hemicellulases, which can degrade hemicellulose into smaller polymers and sugars. Xylanases (endo-1,4-β-xylanases, EC 3.2.1.8) are one of the most representative enzymes within this group, classified as endo enzymes that catalyze the hydrolysis of the β-1,4 linked chains of xylose to produce small XOs [40,73,76,77]. Xylanases are grouped as glycoside hydrolases (GH) in the families of glycosidases 10 (GH10) and 11 (GH11) [72]. GH10 enzymes have a preference for groups at the terminal of xylan bonds, particularly favoring the reducing end, and can degrade dorsal branches with many substitutions because they have low substrate specificity. On the other hand, GH11 enzymes show a preference for internal xylan bonds and unsubstituted xylan chains, acting primarily on the xylose unit in the center of the oligosaccharide chain, hydrolyzing only xylan [78].

Some other enzymes of the hemicellulases group can be used in the production of XOs in conjunction with xylanases, aiming to increase the yield and specificity of production [79,80]. Arabinofuranosidases (EC 3.2.1.55) can act on polysaccharides containing arabinose, catalyzing the hydrolysis of the glycosidic bond of the nonreducing terminal, generating arabinoglucoxylans, arabinoxylan, and arabinans [81,82]. Feruloyl esterases hydrolyze the ester bond between the arabinose and ferulic acid substituents in xylose, while acetyl esterases hydrolyze acetyl substituents in xylose [40]. The β-xylosidases (EC 3.2.1.37) have the ability to hydrolyze xylobiose at the nonreducing end, as well as soluble XOs, releasing xylose. However, xylose is a monosaccharide that decreases the prebiotic effect of XOs, which is why β-xylosidases are not usually used in the production of XOs [83,84,85].

**Table 3:** Enzymes used in xylooligosaccharides production from different agro-industrial residues.

Residue	Enzyme	Hydrolysis Condition	Yield	Reference
Corn cob	Endoxylanase	50 °C, pH 5.4, 14 h	10.2 mg/mL	[86]
Nervosum grass	Endoxylanase from *Trichoderma viridae*	40 °C, pH 5.11, 16.5 h	0.11 g/g	[87]
Corn cob	Endoxylanase	40 °C, 140 rpm, 7 d	150 mg/g	[88]
Bean culm			105 mg/g	
Bagasse			133 mg/g	
Mahogany wood	Xylanase	50 °C, pH 5.0, 12 h	572.00 mg/g	[89]
Mango wood			504.00 mg/g	
Wheat straw	Endo-β-1,4-xylanase	50 °C, pH 4.8, 150 rpm, 48 h	7.10 mg/mL	[90]
Arecanut husk	Endo-β-1,4-xylanase	50 °C, pH 5.0, 24 h	350.00 mg/g	[91]
Rice husk	Endo-β-1,4-xylanase	60 °C, pH 6.0, 9 h	17.35 mg/mL	[75]
Maize straw	Xylanase	50 °C, pH 5.0, 200 rpm, 7 h	670.00 mg/g	[92]
Poplar	Xylanase and celullase from *Aspergillus orizae*	50 °C, pH 5.0, 200 rpm	16.9 g/kg	[93]
Sugarcane bagasse	α-l-arabinofuranosidase, endo-1,4-xylanase, and feruloyl esterase	50 °C, pH 5.0, 48 h	10.23 mg/mL	[73]
Coffee husk			8.45 mg/mL	
Coffee peel	Endo-β-1,4-D-xylanase	40 °C, 24 h	407.5 mg/g	[94]
Beech wood	Crude xylanase	40 °C, pH 6, 24 h, 180 rpm	10.1 mg/mL	[95]
Sugarcane bagasse	Endoxylanase (*rHlxyn11* A)	40 °C, pH 5.5, 180 rpm, 96 h	587.30 mg/g	[96]
Corn cob powder	Immobilized xylanase	40 °C, pH 5, 2 h	126.7 mg/g	[97]
Milled rice straw	Immobilized xylanase	50 °C, pH 7, 5 h	143 mg/g	[98]
Milled corn cob			152 mg/g	
Birch wood	Recombinant xylanase (*reBlxA*)	40 °C, pH 6.0, 24 h	3.02 mg/mL	[99]
Pineapple bagasse	Recombinant xylanase	50 °C, pH 5.2, 180 min	2.70 mg/mL	[100]
			2.31 mg/mL	
Kenaf stem	Recombinant xylanase and arabinofuranosidase (*Xyn2:AnabfA*)	40 °C, pH 4.0, 48 h	351.46 mg/g	[101]
Corn cob	Recombinant xylanase LC9	40 °C, 200 rpm, 72 h	6.91 mg/mL	[74]
Beech	Xylanase (*XynB*) and α-glucuronidase (*AguA*)	80 °C, pH 7.0, 6 h	17.29 mg/mL	[102]
Soybean fiber	Recombinant endo-1,4-xylanase (*xynC*) and α-l-arabinofuranosidase (*abfB*)	50 °C, pH 4.7, 10 h	37.25 mg/X2	[80]
			288.6 mg/g X3	
			143 mg/g X4	
Sugarcane bagasse	Recombinant endoxylanase modified from GH10 xylanase of alkaliphilic *Bacillus Halodurans*	60 °C, pH 7.0, 24 h	4.66 g/L	[103]
Sugarcane bagasse	Recombinant xylanase (*XynA*)	60 °C, pH 5, 180 rpm, 6 h	33.32 g/L	[104]
Wheat bran	Recombinant endo-β-1,4-xylanase (*Baxyl11*)	44.3 °C, pH 7.98, 12 h	5.3 mg/mL	[105]
Bamboo hemicellulose (BCH)	Recombinant xylanase (*HoXyn10*), α-glucuronidase (*AnGus67*), and α-L-arabinofuranosidase (*AnAxh62A*)	50 °C, 24 h	7.08 g/L	[106]
Corn cob	Recombinant endoxylanases and exoxylanases (GH10 and GH11)	50 °C, pH 6.0, 48 h.	115 mg/g	[62]
Sorghum stalk	Immobilized recombinant endo-1, 4-β-D-xylanase (XynC)	50 °C, 72 h	40.81 mg/g	[107]
Sugarcane bagasse			38.43 mg/g	
Hydrothermal liquor of Eucalyptus wood chips	Immobilized recombinant xylanase (MpXyn10)	50 °C, pH 5.0, 3 h	0.9 mg/mL	[108]

Several studies have demonstrated the ability of xylanases to obtain XOs from agro-industrial residues. Brienzo et al. [109] and Khat-udomkiri et al. [75] demonstrated that reaction time and enzyme concentration influenced XOs yields, regardless of whether unpurified enzyme extract or commercial enzyme was used. Decreasing the enzyme concentration resulted in decreased hydrolysis, while increasing the reaction time led to higher concentrations of xylobiose and xylose and decreased xylotriose concentration. The production of XOs can be achieved through simultaneous enzymatic production and enzymatic hydrolysis [88]. Li et al. [110] successfully produced xylotetrose (X4) and xylopentose (X5) from various substrates using the hydrolytic capacity of xylanase produced by *Streptomyces rameus* L2001 in submerged fermentation.

Some techniques have also been studied to increase the enzymatic efficiency of XOs production, such as immobilization and recombinant production. Li et al. [111] (2014) immobilized a recombinant xylanase from *Trichoderma reesei* in ethylene glycol methyl ether 5000 (MPEG5000) in an aqueous biphasic MPEG5000/sodium citrate system and observed a higher yield of XOs production from birch wood compared to the free enzyme. Additionally, Rajagopalan et al. [112] demonstrated that immobilizing endoxylanase in calcium alginate resulted in a more specific production of xylobiose and xylotriose. Furthermore, Purohit et al. [98] developed a magnetic cross-linked xylanase immobilized by using *Acinetobacter pitti* MASK 25 and achieved specific production of XOs (xylopentose and xylohexose) using pretreated rice straw and corn cob. The magnetic immobilized xylanase promoted over 60% of xylan conversion into XOs.

### 3.4. Biorefinery Concept Applied to Xylooligosaccharides Production

XOs are a high-value material, and their production from agricultural by-products is a biorefinery concept, as it aims to maximize the utility and value of biomass by its conversion into various products [9]. It is a promising strategy in the production of high-value-added compounds from the hemicellulosic fraction of biomass, promoting the sustainability of the biorefinery model in terms of circular bioeconomy [42]. Integrated lignocellulosic biomass biorefineries can develop bioprocesses to generate bioproducts such as biofuels, bioenergy, enzymes, and chemicals of commercial interest [113]. Cellulose, hemicellulose, and lignin, which are the main components of lignocellulosic biomass, can be used as feedstocks for biorefinery products. Together with XOs, it is possible to produce ethanol, butanol, acetic acid, and citric acid [114].

Some studies have reported XOs production in combination with other products. In a study by Zhang et al. [115], maleic acid was applied to the pretreatment of corn cob for the production of XOs, increasing the efficiency of enzymatic hydrolysis and resulting in a glucose yield of 87.5%. The production of xylanases and other enzymes and the application of enzyme complexes in the production of XOs through the enzymatic hydrolysis of sugarcane bagasse xylan was performed in another study [5]. It was also reported that the integrative biorefinery process for the coproduction of value-added products (gluconic acid and XOs) of a pretreatment method is a good alternative for the integral utilization of sugarcane bagasse [41].

Biorefineries help to reduce the emission of greenhouse gases and generate by-products with added value in a closed production cycle, contributing to the development of sustainability and bioeconomy. However, studies are still needed to advance new technologies in the deployment of biorefineries on a large scale [116].

## 4. The Market of Xylooligosaccharides Prebiotics

### 4.1. Current Market of Xylooligosaccharides

The global scenario and market potential of prebiotics have shown that the oligosaccharide market is increasing promptly, reaching a market size of USD 3.3 billion in 2016 and an expectancy of growth by 8.7% (compound annual growth rate—CAGR) by 2026, with profits up to USD 9.4 billion, twice the estimated market in 2020 (USD 4.5 billion) [117]. The food industry is the first consumer of the prebiotic market due to the addition in formulation, representing 82% of the market [118]. According to the lower requirement to achieve the prebiotic effect, XOs (dose given of 1.4–2.8 g/day) are considered the most competitively priced prebiotic, with a final cost of less than USD 0.1 per dose [34].

The XOs market has increased in the last two decades, since it seems more efficient in boosting and improving the gut microbiota balance and reducing proinflammatory responses than fructooligosaccharides in diet supplementations, potential features for immunity enhancement and cardiovascular and inflammatory diseases [38]. According to the Market Study Reports, the use of XOs is not exclusively in the food industry; nowadays, XOs can also be applied as an immune-stimulating agent in medicine and as an antioxidant agent, nutraceutical, and cosmetic in the pharmaceutical industry [40]. However, XOs application in food products is suitable due to their high stability, acid pH resistance, absorption, digestion, and fermentation of single-cell fatty acids [119]. 

The market price for XOs prebiotics can vary from USD 25 to 50/kg according to their purity, and the minimum selling price is USD 3430–7500, 4030–8970, and 4840–10,640 per metric ton for 80, 90, and 95% purity [120]. The purity of XOs depends on the presence of remaining compounds after processing, such as glucose and xylose, which affect the calorific value and sweetness. Thus, the purity may affect the prebiotic effect [24]. The processes for XOs extraction include autohydrolysis, chemical hydrolysis, enzymatic processes, and combinations among them. A simple extraction process can reach yields of 15.4 to 66% of XOs from raw materials before the purification steps [121]. The minimum daily dose of this prebiotic is about 1.4–2.8 g, making this prebiotic competitive among others due to the price per dose [34,122]. Although there is no XOs daily intake restriction, 5 g per day is the maximum recommended; an excess is not harmful but can cause gases and discomfort [123]. 

Since the market is still expanding, a lucrative value of USD 7.3 billion is estimated by 2023, with an increment projection of USD 130 million by 2025. In 2018, the growth rate was circa 4.1%, and by 2023, it is expected to be 5.3% [38,124]. This prebiotic alternative is still an attractive market and an added-value product since it can be easily produced from hemicellulose residues such as rice straw, wheat straw, and sugarcane [9,55]. 

Currently, the Asiatic market is the highest consumer, especially Japan, the leading country [125]. Meanwhile, China produces 224.9 million tons of corn annually, mainly used for XOs production. Using XOs in animal nutrition, on the other hand, is another alluring market. The benefits of XOs for improving gastrointestinal health can be found in livestock formulations for animal nutrition, including those for cattle, poultry, horses, and swine. Given this, Future Market Insights (FMI) predicted that the market would grow by 5.6% in 2022, with sales of about 6 MT (volume) [126].

### 4.2. Challenges of Xylooligosaccharides for the Food Industry

There are several crucial challenges for the successful integration of XOs as a functional food supplement in the food industry. These challenges are currently being addressed to enable the incorporation of XOs into innovative and healthier food products [1,127,128]. Some of the common challenges are as follows:

(a) Consolidation of scientific studies: While XOs show potential health benefits as prebiotics, further research is needed to establish the specific mechanisms of action, optimal dosage, and potential interactions with other food components. It is essential to have a solid scientific foundation regarding the effects of XOs as prebiotics.
(b) Production costs and availability: The cost and availability of XOs can pose a significant challenge for the food industry. The production of XOs involves enzymatic processes and may require specific raw materials, such as lignocellulosic biomass. Developing cost-effective and sustainable production methods for XOs is necessary to make them economically viable for widespread use in the food industry.
(c) Organoleptic characteristics and stability: XOs, like other prebiotic ingredients, may have unique flavor and texture characteristics that could impact the sensory profile of food products. Ensuring that XOs do not negatively affect the taste, texture, or overall quality of food products is crucial for consumer acceptance. Moreover, XOs can undergo changes during processing and storage, which may affect stability and shelf life.
(d) Quality standards: The food industry must comply with regulatory standards and requirements related to the use of XOs as a food ingredient. Regulatory bodies may have specific guidelines and limits on the concentration and labeling of XOs in food products. Adhering to these standards is necessary to ensure the safe and appropriate use of XOs in food applications.
(e) Consumer acceptance: XOs may be relatively new to the market, and consumers may have limited knowledge about their benefits and potential uses. Educating consumers and raising awareness about XOs as a functional food ingredient is crucial for promoting acceptance and adoption. Creating the right conditions based on solid scientific evidence to support the benefits of XOs is important for building consumer confidence, obtaining regulatory approval, and facilitating the seamless integration of XOs into the food market.

## 5. Patents and Innovation in Technical Development

**Figure 2:**
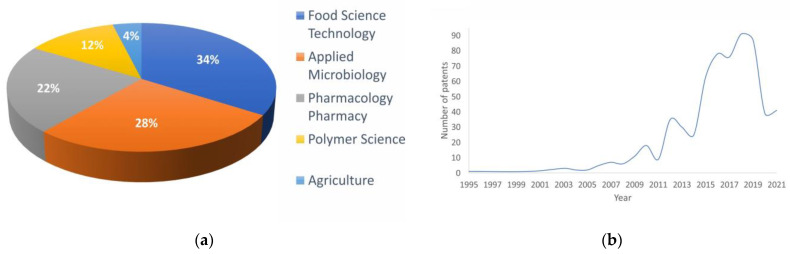
(**a**) Fields of innovation of registered patents in Derwent database; (**b**) number of papers registered annually from 1995 until today.

The production of XOs has been recorded since 1995; according to the Derwent database, there are currently 659 registered patents in different fields (Figure 2). Oji Paper cO, Shandong Longlive Bio Technology Co, Ltd., Yucheng, Shandong, China, and the University of Nanjing are the top three assignees. The most significant XOs producer is Shandong Longlive Biotechnology, located in China, who had the generally recognized as safe (GRAS) certification granted in 2013, as well as Prenexus Health from the USA.

The patent granted to Towa Kasei Kogyo KK was mainly about the methods for producing this prebiotic [129]. The application and innovations only started in 2000, when Suntory Holdings Ltd. (Osaka, Japan) patented agents, among them xylobiose, to lower ammonia in blood and hyperammonemia. The top 10 patents, according to citation and relevance of the database, are from 2012 to 2018. In 2015, Beijing Dongfang Xingqi Food Ind and Tech (Beijing, China) patented a nutritional formulation helpful in improving gastrointestinal tract function; the composition of this formulation contained microcrystalline cellulose, probiotics, XOs, whey protein, and plant extract [130]. Shaoxing Shangyu Hongsheng Technology company (Shaoxing, China) patented another food formulation for enhancing immunity by adding mushroom extracts [131].

Two products were also patented in 2015; the first was by Anhui Yunjiuchang Group Co., Ltd. (Anhui, China), who released a sesame white wine with hepatoprotective properties using XOs as a prebiotic, rice husks, sesame, and plant extracts [132]. The second was a fertilizer for tobacco that included XOs and growth promoters such as potassium tripolyphosphate, citric acid, abscisic acid, and fermented extracts, which was used as a base reference to develop a complete nutrition foliar fertilizer for companies such as Liaoning Agric Sci Acad [133].

In 2014, the University of Jiangnan had a patent granted for preparing XOs and lignin using microcrystalline cellulose to harness agricultural crop straws [134]. In 2013, products and methods for biscuit preparation were also developed, patented, and well cited. The Institute of Agricultural Products, Processing, and Nuclear Agriculture of Hubei [135] developed this product for dietary supplement innovations such as gluten-free and digestive products [136]. In 2012 and 2011, XOs were considered a potential ingredient for addition to animal feed [137] and in alcohol production [138].

Recent innovations and research on XOs have focused beyond the prebiotic qualities that have attracted attention to other potential uses and characteristics of XOs. Recent research on the possible antitumoral action of XOs shows a decrease in the activity of colorectal cancer cell lines [20] and acute lymphocytic leukemia cell lines [139], as well as antiaging effects [140].

In parallel, the incorporation of XOs in biomaterials was also successfully demonstrated. According to Neves et al. [141], XOs can be used as carriers of bioactive compounds. They obtained promising results when XOs nanoparticles were formed to carry a natural blue colorant, genipin. The application as a coating agent was also successfully demonstrated in fruits such as blueberries [142].

Due to their effectiveness as a sugar replacement, moisture retention, heat resistance, and many other characteristics, XOs are still a trend in food composition. However, there are still a few desirable characteristics being studied to create the ideal XOs prebiotic, including the absence of undesirable effects like the gas produced for the metabolism of helpful bacteria, high molecular weight for persistence through the colon without being absorbed, viscosity, stability and storage, and sweetness. Those improvements are desired since the food industry accounted for approximately 49.61% of the XOs market demand in 2017, along with the pharmaceutical sector (25.39%) and food and beverage formulation (23.18%).

Another critical matter to optimize is the industrial scale to produce from an economic point of view, as well as to validate all new properties and characteristics through animal models or human clinical trials since studies of new antitumoral or cytotoxic effects are increasing.

## 6. Conclusions

Prebiotics have become a necessary food supplement in recent years, improving health and nutrition. XOs have a high prebiotic potential compared to other nondigestible oligosaccharides, showing various nutritional and preventive benefits against various systemic disorders. The use of agro-industrial by-products could boost the industrial production of XOs and make it economically viable. New pretreatment methodologies in the production of XOs are more friendly to the environment, reducing the production of toxic compounds. Due to their beneficial properties and characteristics for health, XOs are quickly positioning themselves in the world market, which may approach USD 10 billion in the prebiotics sector by 2026. Different patents and innovations are being developed for the sustainable increase and production of XOs that can consolidate them as a fixed food supplement in the world market of prebiotics.

## Figures and Tables

**Table 1:** Recent examples of effects of xylooligosaccharides prebiotics in different organisms.

Organism	Prebiotic Formulation	Main Components and Manufacturer	Effects	Reference
Humans	Capsule supplement containing 2 g XOs (2.8 g of 70% XOs)	Not provided (purchased from Shandong Longlive Bio-Technology Co., Ltd., Yucheng, Shandong, China)	Modifications on gut microbiota in both healthy and pre-diabetes mellitus subjects (shifts of 4 bacterial taxa associated with the condition)	[19]
Humans	2.2 or 4.8 g/d arabino-xylan-oligosaccharides (AXOs)	Not provided (purchased from Kellogg Company, Battle Creek, MI, USA)	Selectively increased fecal bifidobacterial and postprandial ferulic acid concentrations	[23]
Humans	Prebiotic berry juice (50 mL, XO quantity not provided)	Not provided (purchased from AKK Formula^TM^)	Improved levels of skin brightness, moisture, elasticity, spots, and brown spots—mechanisms not detailed	[20]
*Megalobrama amblycephala* (Fish)	1.0% of XOs supplemented in the daily diet	Not provided (purchased from Yuanye Biotechnology Co., Ltd., Shanghai, China)	Improved growth performance and glycolipid metabolism (upregulating glucose transport, glycolysis, glycogenesis, pentose phosphate pathway, and fatty acids β-oxidation; downregulating gluconeogenesis and fatty acid biosynthesis)	[25]
*Oreochromis niloticus* (Fish)	5.0% of XOs supplemented in the daily diet	Not provided (purchased from Cargill, Wayzata, MI, USA)	Enhanced production of glutathione-related proteins—antioxidation and detoxification effects	[26]
Broiler Chickens	0.005% or 0.01% of XOs supplemented in the daily diet	Not provided	Enhanced production of SCFA through increased cecal fermentation; improvements in body weight	[27]
Broiler Chickens	Dietary supplementation with 150 mg/kg XOs	Xylobiose, xylotriose, and xylotetraose (purchased from Zhengzhou Yicong Biotechnology Co., Ltd., Zhengzhou, China)	Increased villus height of duodenum, jejunum, and ileum, and VH/CD (villus height/crypt depth) ratio of jejunum; enhanced nitrogen metabolism and reduced fecal ammonia release	[28]
Pigs	Dietary supplementation with 100, 250, and 500 g/t XOs	Xylobiose, xylotriose, and xylotetraose (purchased from Shandong Longlive Biotechnology Co., Ltd., China)	Reduced pathogenic bacteria (*Proteobacteria* and *Citrobacter*) and enhanced beneficial bacteria (*Firmicutes* and *Lactobacillus*); decreased level of 1,7-heptane diamine and increased concentrations of acetic acid, straight-chain fatty acids, and total SCFAs in the intestine	[29]

**Table 2:** Composition, types of pretreatments, and yields derived from agro-industrial residues used in xylooligosaccharides production.

Residue	Composition	Pretreatment	Yield	Reference
Sugarcane bagasse	Glucan 37.61%	Hydrothermal	XOs: 67.12%	[59]
	Xylan 21.87%			
	Lignin 20.60%			
Sugarcane bagasse	Cellulose 38.9%	Hydrothermal and organosolv	Conversion of 90% into XOs	[60]
	Hemicellulose 28.2%			
	Lignin 19.7%			
Sugarcane bagasse	Glucan 43.49%	Hydrothermal	XOs: 50.53%	[61]
	Xylan 22.68%			
	Lignin 20.75%			
Corn cob	Glucan 37.5%	Hydrogen peroxide-acetic acid	XOs: 58.3 g of XOs from 1 kg of corn cob	[44]
	Xylan 32.9%			
	Lignin 13.7%			
Corn cob	Glucan 30.3 ± 0.9	Alkali and hydrothermal	Alkali: 59 mg of XOs; hydrothermal: 115 mg of XOs per gram of initial biomass	[62]
	Xylan 25.8 ± 0.4			
	Lignin 20 ± 1			
Corn cob	Glucan 33.1%	Gluconic acid	180 g of XOs per 1 kg of corn cob	[63]
	Xylan 32.8%			
	Lignin 19.0%			
Wheat straw	Glucose 41.17 ± 2.04	Hydrothermal	N.C.	[64]
	Xylose 25.73 ± 1.28			
	Lignin 20.13 ± 1.01			
Poplar	Glucan 44.0%	Sodium chlorite and acetic acid	4.0 g of XOs and 6.6 g of xylose per 100 g of poplar	[65]
	Xylan 18.4%			
	Lignin 26.3%			
Bamboo shoot shell	Glucan 38.2 ± 1.0	Hydrothermal	6.6 g of XOs per 100 g of bamboo shoot shell	[66]
	Xylan 25.7 ± 0.1			
	Lignin 25.2 ± 0.2			
Cassava peel-based	Cellulose 26.07 ± 2.46	Sodium hydroxide	24.21 mg of XOs per 1 g of pretreated biomass	[67]
	Hemicellulose 13.09 ± 0.62			
	Lignin 38.30 ± 2.24			
Poplar sawdust	Glucan 46.9 ± 0.3	Acetic acid	7.2 g XOs per 100 g of poplar sawdust	[68]
	Xylan 18.6 ± 0.3			
	Lignin 28.3 ± 0.6			
Peach palm—inner sheath	Cellulose 34.2 ± 4%	Sodium hydroxide	XOs: 50.1%	[69]
	Hemicellulose 19 ± 2%			
	Lignin 23 ± 2%			
Peach palm—peel	Cellulose 36.0 ± 4%	Sodium hydroxide	XOs: 48.8%	[69]
	Hemicellulose 20 ± 4%			
	Lignin 20 ± 3%			
Pineapple peel waste	Cellulose 20.9 ± 0.6	Hydrothermal	XOs: 25.7 ± 0.4 g/100 g of xylan; xylose: ~91.3%	[70]
	Hemicellulose 31.8 ± 1.9			
	Lignin 10.4 ± 1			
Birch sawdust	Glucan 46.6	Hydrothermal	XOs: 46.1%	[71]
	Xylan 22.4			
	Lignin 23.7			
Rice husk	Cellulose 28.6%	Microwave	Arabinoxylan: 9.01 g/100	[53]
	Hemicellulose 28.6%			
	Lignin 24.4%			
Sugarcane bagasse	Cellulose 43.6%	Microwave	290.2 mg/g	[57]
	Hemicellulose 33.5%			
	Lignin 18.1%			
Wheat straw	Glucan 33.2% ± 0.4	Microwave-assisted deep eutectic solvent	72.7 g/kg wheat straw	[58]
	Xylan 19.7% ± 0.1%			
	Lignin 21.1% ± 0.1			

N.C. not calculated.

## Data Availability

Data is contained within the article.
